# The cost of the training and supervision of community health workers to improve exclusive breastfeeding amongst mothers in a cluster randomised controlled trial in South Africa

**DOI:** 10.1186/s12913-020-4913-4

**Published:** 2020-02-03

**Authors:** Gavin George, Takunda Mudzingwa, Christiane Horwood

**Affiliations:** 1 Health Economics and HIV and AIDS Research Division (HEARD), University of KwaZulu-Natal, Durban, South Africa; 2 Centre for Rural Health, University of KwaZulu-Natal, Durban, South Africa

**Keywords:** Costing, Community health workers, Community case management, Continuous quality improvement supervision, Exclusive breastfeeding, South Africa

## Abstract

**Background:**

Interventions targeting community health workers (CHWs) aim to optimise the delivery of health services to underserved rural areas. Whilst interventions are evaluated against their objectives, there remains limited evidence on the economic costs of these interventions, and the practicality and value of scale up. The aim of this paper is to undertake a cost analysis on a CHW training and supervision intervention using exclusive breastfeeding rates amongst mothers as an outcome measure.

**Methods:**

This is a retrospective cost analysis, from an implementer’s perspective, of a cluster randomised controlled trial investigating the effectiveness of a continuous quality improvement (CQI) intervention aimed at CHWs providing care and support to pregnant women and women with babies aged < 1 year in South Africa.

**Results:**

One of the outcomes of the RCT revealed that the prevalence of exclusive breastfeeding (EBF) significantly improved, with the cost per mother EBF in the control and intervention arm calculated at US$760,13 and US$1705,28 respectively. The cost per additional mother practicing EBF was calculated to be US$7647, 88, with the supervision component of the intervention constituting 64% of the trial costs. In addition, women served by the intervention CHWs were more likely to have received a CHW visit and had significantly better knowledge of childcare practices.

**Conclusion:**

Whilst the cost of this intervention is high, adapted interventions could potentially offer an economical alternative for achieving selected maternal and child health (MCH) outcomes. The results of this study should inform future programmes aimed at providing adapted training and supervision to CHWs with the objective of improving community-level health outcomes.

## Background

Community health workers (CHWs) have the potential to address the barriers encountered by women and children in accessing maternal and child health (MCH) services, particularly in underserved rural areas where the shortage of professional health workers is most profound [1]. CHWs can increase coverage of maternal and child services in African settings, leading to improved maternal and child health outcomes [2, 3]. In 2010, the South African government proposed to re-engineer the provision of primary health care by focusing on a small number of key priorities, including the training and deployment of a generalist CHW that would become a recognised cadre within the national health system [4]. Ahead of implementation of the PHC re-engineering, a 2011 audit estimated that 72,000 CHWs were active across the country [5]. The majority were single purpose workers, linked to NGO-driven HIV and TB programmes offering a plethora of services but without a formal scope of practice.

CHWs focus primarily on community engagement, risk identification, and referral to public health facilities [6]. The comparative advantage of utilising CHWs remains that they are from the community and are therefore closer to the patient and their families and more accessible compared to facility based health personnel [7, 8]. CHWs can assist in bridging the gaps present in the delivery of integrated care at the health facility level [9] with several studies highlighting the potential role of CHWs in increasing access to health care for individuals living in underserved locations in South Africa [10, 11].

Although CHWs may be considered a less expensive alternative to other professional health cadres, with regard to salary, they should not be seen as a cheap solution for weak health systems as they require support and supervision to be effective and sustainable [1]. Literature has pronounced on this health cadre’s lack of supervision as a key impediment to optimising their utility [1, 12], with further studies confirming the lack of support afforded to CHWs by the health system [8] as well as how providers readily offload responsibilities to them without assuming a supervisory role [10]. Many health facility managers similarly have not assumed their supervisory role and provided inadequate support to CHWs [12]. The effectiveness of CHWs therefore depends on the presence of regular [13] and skilled supervision [14]. Thus, achieving optimal outcomes through the utilisation of CHWs relies on cost-effective strategies that provide quality management and supervision [15].

Research suggests that supervision and constant quality management of the services provided by CHWs will enhance community health outcomes [1, 16, 17]. However, little is known about the costs of such interventions aimed at CHWs. The objective of this study is, therefore, to cost an intervention using the WHO adapted continuous care management (CCM) training materials and a continuous quality improvement (CQI) mentoring and supervision approach targeting CHWs in South Africa [18]. The intervention aimed to achieve a number of objectives as described elsewhere [19]. This costing analysis will express outcomes as natural units represented by the number of women practicing exclusive breastfeeding (EBF) for the first 6 weeks. The number of women practicing EBF is referred to as measuring an outcome expressed in natural units because it describes direct changes in a health state that an intervention introduces [20]. We assume a direct causal relationship between the activities of CHWs and the change in the number of women practicing EBF. EBF is a beneficial practice that reduces incidence of infant and child diarrhoea, hospitalisations for illness, diarrhoea mortality, and all-cause mortality [21]. If implemented globally at near universal levels (> 90%), optimal breastfeeding practices could reduce global child deaths by more than 800,000, making breastfeeding the most effective preventive intervention to improve infant mortality [21]. Being a beneficial practice, establishing the costs of an intervention aimed at increasing EBF rates would present valuable data for future interventions and programmes aimed at both improving maternal and child health (MCH) outcomes and establishing their cost-effectiveness.

## Methods

### Study setting and intervention

A cluster randomised controlled trial (RCT) investigated the effectiveness of a CQI intervention amongst CHWs providing home-based education and support to selected households, including pregnant women and mothers, in the Ugu Health District in the KwaZulu-Natal (KZN) province, South Africa, between May 2012 and November 2013 [19]. The RCT aimed to measure if the intervention was successful in; 1) improving household caregiving practices including breastfeeding practices, 2) improving uptake of MCH services, and 3) developing clear referral pathways between the community and the local primary healthcare clinic [19]. The RCT consisted of 150 CHWs randomly allocated to standard of care (SOC) and intervention arms (*n* = 75 in each arm). Thirty CHW supervisors were randomly selected from 32 CHW supervisors in the district, and allocated to the intervention or control arms. Four CHWs were randomly selected from the CHWs supervised by each participating CHW supervisor to form a quality improvement team. CHWs were allocated to control or intervention arms based on the allocation of their supervisor. The intervention arm consisted of 15 quality improvement teams comprising five CHWs, including one senior CHW who functioned as the team supervisor. CHWs in the SOC arm received the standard 10-day KZN Department of Health (DoH) training on community-based care for women and infants prior to study initiation. In the intervention arm, CHW received the standard 10-day KZN DoH training, together with an additional 2-week training on WHO Community Case Management (CCM) prior to the study initiation. CCM training included interactive modules focusing on breastfeeding counselling and supporting breastfeeding in the households during antenatal and postnatal visits, using theory, role-plays and clinical demonstrations to develop CHWs skills. CHWs in the intervention arm also received fortnightly CQI mentoring sessions held over 12 months, these were preceded by a 3-month lead-in phase to establish mentoring processes. In addition, quarterly learning sessions took place over the 12-month CQI mentoring period. Quality mentors, who were registered nurses employed by the project and experienced in the use of quality-improvement methods [19], facilitated both the fortnightly mentoring and quarterly learning sessions. This RCT and setting is described in further detail elsewhere [19].

### Intervention outcome

In total, 736 mothers received a CHW visit at baseline, and 606 at follow up [19]. This cost analysis focuses on mothers of infants > 6 weeks (629 at baseline and 531 at follow up) as illustrated in Table 1.

**Table 1:** Reported infant feeding practices amongst participating mothers at baseline and follow-up household surveys

Baseline			Follow up		Change
	N (%)	OR (95% CI)	N (%)	OR (95% CI)	OR (95% CI)
Amongst mothers of infants > 6 weeks					
EBF for first 6 weeks of life					
Control	226/312 (72.4)	Reference	181 / 278 (65.1)	Reference	
Intervention	207/317 (65.3)	0.74 (0.49–1.1), *p* value = 0.13	194/253 (76.7)	1.7 (1.1–2.7), *p* value =0.02	2.3 (1.4–4.0), *p* value = 0.001

Source: [19]

Overall, mothers served by CHWs allocated to the intervention arm were more likely to receive a visit during pregnancy and the postnatal period. Additionally, mothers served by CHWs in the intervention arm had higher maternal and child health knowledge scores and were more likely to have disclosed their HIV status to the CHW. These results and their significance are detailed elsewhere [19].

### Costing

The aim of this economic evaluation was to compare costs and selected outcomes of this intervention. All costs were identified, measured, and valued from a provider perspective. Data were obtained from project budgets and expenditure reports [22]. Additional data, pertaining to donations were obtained from interviews. In estimating resource use and the economic costs incurred in the RCT, we adopted a direct measurement micro-costing approach [23]. The costing considered both financial and economic costs, where financial costs were the actual expenditures incurred in the purchase of items, and economic costs included the opportunity cost of resource utilisation [23].

Costs are expressed in the final year of the intervention thus, all costs were expressed in 2013 prices, collected in local currency (South African Rands, ZAR) and converted to United States Dollars (US$) at an average exchange rate in 2013 of US$1 = R9,66 [24]. Costs were classified as capital or recurrent and captured across three major categories; CHW supportive supervision, CHW outreach activities and CHW training. These categories, associated activities and the resource inputs are listed in Table 2.

**Table 2:** Cost categories and resource inputs for the intervention arm

Costing Categories	Activities	Resource Inputs
Training	WHO Community Case Management (CCM) training	Catering
		Training Venue
		Transport
Support and Supervision	Fortnightly Mentoring Sessions Quarterly Learning Sessions Monthly Meetings	Personnel:3 Quality Mentors; 3 Health Facilitators; 1 Administrator
		Transport
		Meeting Venue
		Equipment (Laptop, projector & printer)
		Office space
		Utilities
		Telephone and data costs
		Stationery
Outreach Activities	Home Visits	Personnel: 120 CHWs & 30 supervisors
		Field Materials

The costs of the standard 10-day KZN DoH training provided to all CHW was not included in the analysis. CHW in the intervention arm who received additional training formed the start-up costs of the intervention and were considered as a capital investment and annualized and discounted, with 10 years chosen as the useful life of the start-up period [25]. This once-off training cost included transport, training venue, accommodation and catering costs which were placed under the category of non-recurrent once-off training costs. The training venue did not carry an explicit cost in the context of the intervention, thus a market price for hiring the venue was obtained and this was included in the cost of the training [26].

All post-training activities were included as recurrent costs and captured in the supportive supervision and outreach categories. This included all costs related to the mentoring and supervision of CHWs. These costed items under mentoring and supervision included personnel, transport, equipment and all other costs related to both mentoring and supervision activities.

Capital costs, within the supportive supervision category included items such as computers and furniture, and other items whose useful life was more than a year. Items whose useful life was more than a year but cost US$100 or less were classified as recurrent costs [27]. Capital costs were calculated using a discount rate of 3% and annuitized over a five-year period, as per WHO recommendations [28]. Furniture, including desks, chairs and a filing cabinet, already existed and was utilised and therefore not explicitly purchased for the project, thus indicative costs were obtained from furniture retailers selling similar items. Overhead costs included office rental and utilities. The project was not charged for either, therefore an annual rental and utility cost of the building space was obtained by estimating the annual price charged for renting and expenditure on utilities for a similar sized unfurnished office space [29]. These costs were split across the two arms.

Recurrent costs included labour, which consisted of five personnel types. Three quality mentors, who each received a monthly salary, facilitated the training, supervision and monitored the entire scope of activities undertaken by the CHWs in the intervention arm [18, 19]. Quality mentor’s time allocated to facilitating the two-week training represented a small portion of the overall time spent on the project by the quality mentors, with their salaries therefore allocated to the supervision category only. In the control arm, supportive supervision was provided by three health facilitators who were enrolled nurses employed on a full time basis and earning an annual median salary of approximately USD21 305,38 [30] compared to a Quality Mentor earning an annual salary of USD56 236,23. Assuming that the health facilitators allocated 10% of their time to CHW activities, it is then estimated that their cost with regards to the control arm is USD2 130, 50 per facilitator. Health facilitators in the control arm met with CHWs on a monthly basis, with no costs incurred. There was one administrator, whose cost was calculated by estimating the percentage of time spent on project related activities multiplied by their full salary [23]. It was estimated that the administrator spent 15% of their time on project related activities, which was allocated to the support and supervision category [22]. The administrator’s salary apportionment was split across the two arms. Other recurrent costs included stationary and supplies, telecommunications and internet data. One quality mentor was provided a cellular phone whilst the other two quality mentors utilised a fixed-line telephone situated in the office. The cost of the fixed-line telephones could not be established. It was assumed that all three-quality mentors used their respective phones equally and thus the cellular phone airtime allowance was used to estimate the fixed-line costs.

CHWs in the intervention arm attended 22 fortnightly mentoring and three quarterly learning sessions, with the costs consisting of a transport allowance paid to CHWs and venue hire and catering costs. These costs are aggregated to the activity level.

The final category, CHW outreach, encapsulates the home visits undertaken by the CHWs. This category included the stipends paid to CHWs and the cost of supplies utilised by CHWs during home visits. The monthly stipend of the 15 CHW supervisors and 60 CHWs allocated to the intervention arm are represented in the cost analysis.

### Cost outcomes analysis

A cost outcomes analysis compared the costs and outcomes of the control and intervention arm of the study [23]. The aim of this analysis is to determine both the cost per mother practicing EBF, and cost per additional mother practising EBF achieved from the intervention. The costs incurred within the training, supportive supervising and outreach categories constitute the intervention costs. A total of 375 mothers had been exclusively breastfeeding for the first 6 weeks of life at the follow-up stage of the study, split into 181 in the control and 194 in the intervention arm (See Table 1). The number of mothers practicing EBF in the intervention arm were adjusted up to account for the larger sample size in the control arm. The percentage of mothers in the intervention arm practicing EBF at follow-up was 76,7 vs 65,1 of mothers practicing EBF in the control arm. The adjusted number of mothers practicing EBF amongst a total of 278, with an uptake rate of 76,7%, was calculated to be 213. The following equation established the cost per additional outcome.

$$ \left({C}_1-{C}_0\right)/\left({E}_1-{E}_0\right)= Cost\  per\  additional\ outcome $$

- *C*_*0*_ *is the cost per mother practicing EBF in SOC.*
- *E*_*0*_ *is the number of mothers practicing EBF in SOC.*
- *C*_*1*_ *is the cost per mother practicing EBF in the intervention.*
- *E*_*1*_ *is the adjusted number of mothers practicing EBF in the intervention.*

### Sensitivity analysis

Studies in similar contexts have used a discount rate of 5% [31], and 8% [32, 33]. In acknowledging this variation, a one-way sensitivity analysis was undertaken, using these alternative discount rates [23]. In addition, we undertook sensitivity analysis using a 5 year useful life time horizon for the once-off training. The sensitivity analysis was therefore limited, as other costs were known with a high degree of certainty. A threshold analysis was not undertaken.

## Results

Table 3 presents the costs by input categories. Costs related to CHW supervision dominated the trial costs, constituting 64% of all programme costs, followed by CHW outreach activities which contributed 35% of the total cost.

**Table 3:** Project costs by category

Costing Categories & Inputs (Quantity)	Intervention Arm			Control Arm		
	Costs (US$)	% within inputs	% of total cost	Costs (US$)	% within inputs	% of total cost
**Support Supervision**	**244,597,93**	**100%**	**64%**	**20,433,02**	**100%**	**15%**
Personnel: Quality Mentors (3) / Health Facilitators (3)	168,708,70	69%	44%	6391,61	31%	5%
Administrator (1)	2739,54	1%	1%	2739,54	13%	2%
Mentoring Sessions (22)	51,545,03	21%	13%	–	–	–
Quarterly Learning Sessions (3)	10,302,80	4%	3%	–	–	–
Monthly Meetings	–	–	–	0	0%	0%
Equipment (Laptop, projector & printer)	710,87	0%	0%	710,87	3%	1%
Office space	6972,05	3%	2%	6972,05	34%	5%
Utilities	1612,32	1%	0%	1612,32	8%	1%
Telephone and data costs	747,20	1%	0%	747,20	4%	1%
Stationery	1259,42	1%	0%	1259,42	6%	1%
**Once-off Training**	**1862,52**	**100%**	**0%**	**0,00**	**0%**	**0%**
Combined training costs	1862,52	100%	0%	0,00	0%	0%
**Outreach Activities**	**117,150,62**	**100%**	**35%**	**117,150,62**	**100%**	**85%**
Personnel: CHWs (60 in each arm)	89,440,99	76%	27%	89,440,99	76%	65%
Supervisors (15 in each arm)	26,086,96	22%	8%	26,086,96	22%	19%
Field Materials	1622,67	1%	0%	1622,67	1%	1%
**TOTAL**	**363,611,07**			**137,583,64**		

Costing Categories and totals in bold

The greatest proportion of recurrent costs was personnel, contributing over 80% of the total economic cost of the programme. Quality Mentors make up the highest proportion (44%) of the costs of the intervention. The Quality Mentors, who were highly skilled professional nurses with management experience, were paid market related salaries. The second highest contributor to costs were the CHW’s stipend costs (27%). The high CHW personnel costs are a function of the number of CHWs in the programme rather than a reflection of their skills base. The mentoring and the quarterly learning sessions that CHWs and supervisors attended, contributed a combined 16% and are the third highest contributor as reflected in Table 3. The once-off WHO adapted CCM training was a negligible cost relative to the total cost of the intervention.

### Outputs and costs

Table 4 illustrates the study outcomes and the associated costs. The cost per mother EBF in the control arm was US$760,13 and US$1705,28 in the intervention arm. The cost per additional mother practicing EBF was calculated to be US$7647,88.

**Table 4:** Outcomes and average costs (US$)

Outcomes	Total	Average Costs (US$)
Control Arm Mothers Exclusively breastfeeding for first 6 weeks of life	181	760,13
Intervention Arm Mothers Exclusively breastfeeding for first 6 weeks of life (adjusted)	213	1705,28
Additional Mothers Exclusively breastfeeding for first 6 weeks of life	32	7647,88

### Sensitivity analysis

Discount rates of 5, and 8% were calculated (results not shown). A higher discount rate resulted in a marginally higher cost per additional outcome. Since the change is marginal, the sensitivity analysis indicated that the results were robust to these parameter variations [23]. Additionally, the useful training life was reduced to 5 years from the 10 used in the analysis reported above. Given the relatively low costs attributed to training, this analysis resulted in only a marginal change and is not reported.

## Discussion

This study analysed the costs of implementing a WHO adapted CCM training and a CQI mentoring and supervision intervention, which was successful in increasing EBF rates at 6 weeks post-partum. In this study, the cost per mother practicing EBF in both the control and intervention arms (US$760,13 and US$1705,28) is more expensive when compared the cost per additional mother practicing EBF calculated in other South African studies [32], even after adjusting for different outcomes; cost per increased month of EBF [34]. Although the results reveal that the intervention was relatively costly, there were additional benefits accrued, including the increased number of antenatal and postnatal household visits by CHWs and the proportion of mothers with increased health promotion knowledge [19]. These benefits are very likely to have contributed to improved infant feeding practices and care-seeking behaviours of mothers [19]. A unit value could not, however, be allocated to these benefits. If these benefits could be valued, the cost of the intervention could potentially be apportioned over additional outcomes and not solely to EBF, with the resultant costs potentially decreasing significantly, increasing the likelihood of a cost effective intervention.

Carefully selected, trained and supported CHWs offer a potentially economical alternative for promoting and supporting EBF and other key maternal and child health practices to underserved communities, compared with the reliance on overburdened health facilities [21]. Whilst CHWs may potentially be an economical alternative for promoting EBF, CHWs possess less formal training than facility-based health workers. This results in the need for supervision and support [35] such that, to optimise the utility of CHWs, supervision is required [13, 14].The personnel costs associated with skilled supervision are expensive as revealed by this and other studies [32, 34], and suggests that the costs of skilled supervision adds substantially to the overall costs of community based programmes. However, without supervision, it is unlikely that optimal benefits will be achieved. In this study supervision was conducted by outside facilitators employed by the study, but in a larger scale rollout it would be possible for existing supervisors to adapt this approach to be used during routine visits to CHWs, thereby reducing costs.

Future research needs to determine whether improved EBF rates, and other MCH outcomes, remain attainable with different or lower-level health worker cadres operating as supervisors or less costly training or fewer mentoring sessions. It therefore remains crucial to test alternative supervision models aimed at increasing MCH outcomes using CHWs, whilst costing them. This study therefore adds to a limited body of costing analysis of training and supervisory models targeting CHWs [36].

Further research and analysis on the impact of alternative supervision interventions, combined with an economic evaluation, is essential in assessing whether the added costs are reasonable when compared to the MCH outcomes achieved [32]. A central tenet of any supervisory model remains scalability and sustainability [19].

### Limitations

The standard 10-day KZN DoH training provided to CHWs in both the control and intervention was not costed. These costs would have affected only the cost per mother practicing EBF result, but not significantly, given the proportion of the cost allocation of the intervention training. Furthermore, the 15-month time horizon of the trial may not have been sufficient in terms of realising the study outcomes in their entirety. The initial implementation period of CQI supervision is costly but decreases once CHWs are accustomed to the principles behind CQI management resulting in the tapering off of supervision and the resultant reduction in costs. This highlights the observation that without understanding that supervision is a long-term management intervention, the benefits of CQI may be discounted too hastily [18]. It is important to note that the time between the retrospective cost analysis and the RCT, potentially increased the probability of errors affecting the analysis, primarily due to recall basis [18]. Lastly, this study did not calculate the social costs derived from the increased EBF rates or the opportunity costs of CHWs. These costs are potentially important when determining the true value of an intervention and the outcomes associated with it.

## Conclusion

Whilst CHWs may be a low cost alternative to professional health workers, they require skilled supervision to operate effectively. CHW training and supervision was shown to effectively improve coverage of CHW visits, improve knowledge of mothers about child care practices, as well as leading to improved household breastfeeding practices. The relevance of this study is that it contributes to research on the cost of providing supervision to CHWs; however, more research around this topic is required.

## Data Availability

The datasets used and/or analysed during the current study are available from the corresponding author on reasonable request.

## Abbreviations

CCM: Community case management
CHW: Community health worker
CQI: Continuous quality improvement
DoH: Department of Health
EBF: Exclusive breastfeeding
KZN: KwaZulu-Natal
PHC: Primary health care
RCT: Randomised control Trial
US$: United States Dollars
WHO: World Health Organization
ZAR: South African Rands
